# Autophagy in Cell Survival and Death

**DOI:** 10.3390/ijms24054744

**Published:** 2023-03-01

**Authors:** Jinsung Yang, Wanil Kim, Deok Ryong Kim

**Affiliations:** Department of Biochemistry and Convergence Medical Science, Institute of Health Sciences, College of Medicine, Gyeongsang National University, Jinju 52727, Republic of Korea

Autophagy is a degradative process to remove damaged or unnecessary cellular components, and it has been implicated in many biological processes during cell survival and death [1]. Several factors, such as the availability of nutrients and growth factors, intracellular metabolic situations, and other environmental stressors, could determine the cell fate between survival and death, as shown in Figure 1. Under these conditions, autophagy plays a multifunctional role in cell fate determination. It can promote cell growth under starvation by providing nutrients necessary for survival through the degradation of self-components. Additionally, autophagy can induce cell death under specific conditions, including prolonged starvation and stress, by activating the apoptotic process. The context-dependent activation of autophagy is critical for deciding cell fate. To date, the molecular mechanism of induction of autophagy is relatively well-established, but the mechanisms interacting with autophagy are not fully understood. This Special Issue focuses on the link between autophagy and various biological functions.

Biagioni et al. suggested that the autophagoproteasome is suppressed within the ischemic penumbra [2]. The ischemic penumbra is a region in the brain between the dying part caused by ischemic stroke and the salvageable part that still receives blood supply. The overexpression of HSP70 is a well-established biochemical marker of the penumbra area. The region’s autophagy-related proteins, including LC3, increase to keep the system cleared. Indeed, P20S colocalized with LC3 within autophagoproteasome vacuoles, and these proteins were massively degraded within the penumbra area. This study provides a molecular and spatial understanding of the ischemic penumbra.

Lin et al. showed that fatty acid binding protein 6 (FABP6) regulates cell cycle progression and autophagy in bladder cancer (BC) cells [3]. Knockdown of FABP6 inhibited BC cell growth via regulation of cell-cycle-related protein expressions. It also caused a severe decrease in nuclear receptor PPARs/RXRα and activation of NF-κB signaling. Moreover, in the FABP6-deficient cells, the activated signaling of AKT and mTOR inhibited autophagy. As a result, FABP6 regulates the focal adhesive protein level in BC cells and decreases cancer cell migration. This study suggested that controlling FABP6 can be a novel strategy for the progression of BC cells.

Jalouli et al. stressed the adverse effect of pyrethroid-based insecticides in developing rat ovaries [4]. Allethrin is one of the most commonly utilized pyrethroids. In the paper, the authors intragastrically administered allethrin and assessed the consequences. Allethrin caused structural damage and oxidative stress in ovaries. It also inhibited the expression of PI3K/AKT/mTOR. Moreover, allethrin increased the expression of the autophagy marker, LC3, and proapoptotic marker, caspase-3. In conclusion, exposure to allethrin may cause significant damage to the female reproductive system via the structural and functional alterations in the ovary.

Kim et al. showed that zinc deficiency activates autophagy in hippocampal neuronal cells [5]. Zinc deficiency due to the zinc chelator, N, N, N′, N′-tetrakis (2-pyridyl methyl) ethylenediamine (TPEN), in HT-22 cells promoted the lipidation of LC3 and its subsequent cytotoxicity. Additionally, TPEN treatment increased the expression of several autophagy-related genes and the phosphorylation of AMPK. Moreover, autophagy inhibition reversed TPEN-dependent cell survival. Therefore, this study demonstrated that neural cells are protected by zinc deficiency-induced autophagy.

Jeon et al. researched the role of the cytoprotective function of L-serine in hippocampal neurons [6]. Propionic acid (PPA) decreased cell viability and lysosomal activity in HT-22 cells. Moreover, PPA-induced lipid accumulation was reduced by L-serine. Colocalized lipid droplets and lysosomes were observed regardless of lysosomal activity. L-serine recovered dysfunctional lysosomes induced by PPA and decreased lipid accumulation. In conclusion, L-serine controls lipid metabolism by inducing the lysosomal activity in hippocampal neurons.

Jung et al. proposed that chrysin, a flavonoid compound, induces both autophagy and apoptosis in human mucoepidermoid carcinoma (MC-3) cells [7]. According to the results, chrysin treatment stimulated autophagy via the lipidation of LC3, expression of Beclin-1, and inhibition of the mTOR signal. This chrysin-induced autophagy significantly protected the cells from apoptosis triggered by chrysin via mitogen-activated protein kinases (ERK, JNK, and p38), suggesting that balance between autophagy and apoptosis is critical for maintaining cellular homeostasis and that chrysin has a potential anticancer effect in MC-3 cells.

Lv et al. discussed the role of exogenous hydrogen sulfide (H_2_S) in regulating autophagy in diabetic-related diseases [8]. H_2_S is a signal transducer in many biological functions, including antiapoptosis, antioxidative stress, blood vessel relaxation, blood pressure reduction, and the anti-inflammatory process. Indeed, H_2_S triggered autophagy via the Keap1 [9], AMPK/mTOR [10], PI3K/AKT1 [11], SIRT6/AMPK [12], Nrf2/ROS/AMPK [13], TGFβ1/NF-κB/AKT [14], and BDNF/TrkB [15] pathways in diabetes-related diseases. In conclusion, understanding the role of autophagy regulated by H_2_S may provide a potential therapeutic strategy for diabetes.

Yang discussed autophagic regulation during the viral binding to cell surface receptors [16]. A virus binds to its specific receptors to infect cells. Binding viral particles to their specific receptors on the host cells can regulate several intracellular signalings, including autophagy, during infection. This autophagy activation subsequently triggers the removal of pathogens via lysosomal degradation, but how viral binding to receptors on the cell surface induces autophagy is unclear. Many viruses use CD46, TLRs, and integrins as binding receptors. In the paper, the author summarized some details about how these interactions trigger and activate autophagy and what are determinants for either viral survival or eradication, although further studies are necessary to understand the effect of viral binding in regulating autophagy.

Truzzi et al. suggested that wheat-germ spermidine (SPD) and clove eugenol (EUG) combination supplement (SUPPL) could reduce inflammatory parameters by activating autophagy in intestinal epithelial cells [17]. SUPP ameliorated the inflammation induced by lipopolysaccharide through the activation of AMPK-dependent autophagy. Therefore, this study exhibits the impact of SUPPL on anti-inflammation by targeting autophagy. 

In conclusion, autophagy is a biological response that protects cells from internal or external stimuli and interacts with various cell signaling systems. This Special Issue discussed the role of autophagy in neurons, adipocytes, and the uterus. In addition, it discussed that various stimuli regulate the activity of autophagy. Autophagy can either save or kill cells depending on the cellular condition, but further studies could be necessary for assuring its detailed mechanisms in the future. These studies will shed light on understanding autophagy-dependent cell survival and death in different systems. We hope this Special Issue will be helpful to researchers who study autophagy.

## Figures and Tables

**Figure 1:**
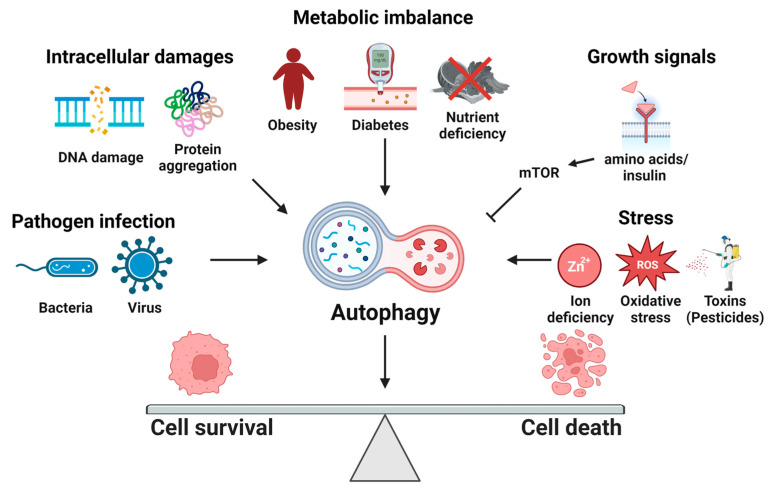
Cell fate can be determined by autophagy regulators under many biological conditions. This figure was created with www.Biorender.com, accessed on 1 March 2023.

## Data Availability

Not applicable.
